# Mutation of the attractin gene impairs working memory in rats

**DOI:** 10.1002/brb3.2876

**Published:** 2023-01-09

**Authors:** Meng‐Qi Liu, Cheng Xue, Xiao‐Hui Li, Hong‐Qun Ding, Meng‐Yu Zhang, Kai Chen, Ying Li, Shu‐Zhan Gao, Xi‐Jia Xu, Wei‐Ning Zhang

**Affiliations:** ^1^ School of Medicine Jiangsu University Zhenjiang Jiangsu Province P. R. China; ^2^ Department of Clinical Laboratory Changzhou Second People's Hospital affiliated to Nanjing Medical University Changzhou P. R. China; ^3^ Department of Clinical Laboratory, Xiangyang First People's Hospital Hubei University of Medicine Xiangyang P. R. China; ^4^ Department of Psychiatry the Affiliated Brain Hospital of Nanjing Medical University Nanjing Brain Hospital Nanjing P. R. China

**Keywords:** attractin (ATRN), mutation, myelin basic protein (MBP), working memory

## Abstract

**Objective:**

Attractin (ATRN) is a widely expressed member of the cell adhesion and guidance protein family in humans that is closely related to cellular immunity and neurodevelopment. However, while previous studies in our laboratory have confirmed the effect of ATRN mutations on long‐term memory, its specific role and the molecular mechanism by which it influences spatial cognition are poorly understood.

**Methods:**

This study aimed to examine the effect of ATRN mutations on working memory in water maze with a novel ATRN‐mutant rat generated by the CRISPR/Cas9 system; the mutation involved the substitution of the 505th amino acid, glycine (G), with cysteine (C), namely, a mutation from GGC to TGC. The changes in myelin basic protein (MBP) expression in rats were also analyzed with the western blot.

**Results:**

The ATRN‐G505C(KI/KI) rats exhibited significant increases in the required latency and distance traveled to locate the escape platform in a Morris water maze test of working memory. In addition, the expression of MBP was reduced in ATRN‐mutant rats, as shown in the western blot analysis.

**Conclusion:**

Our results indicate that ATRN gene mutations may directly lead to the impairment of working memory in the water maze; this impairment may be due to the inhibition of MBP expression, which in turn affects the spatial cognition.

## Graphical abstract

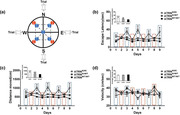

## 1 INTRODUCTION

Attractin (ATRN) is widely expressed in the human body and has been shown to play an important role in mediating cellular immunity and neurodevelopment (Paz et al., 2007; Radhakrishnan et al., 2009). It has a variety of isoforms and two of them are well established: the transmembrane domain protein and the secreted protein. Functional expression of all ATRN isoforms is undertaken by extracellular motifs, including several epidermal growth factor (EGF) domains, one CUB, and one C‐type lectin domain (Duke‐Cohan et al., 1998; Tang et al., 2000). Both the membrane‐bound and secreted isoforms contain two EGF domains and two transparent membrane glycoprotein EGF domains. The membrane‐bound isoform interacts with Sertoli cells and coordinates with receptors (Nakadate et al., 2008), and the secreted isoform consists of glycosylated serum proteins that regulate the distribution of monocytes and the accumulation of T cells (Wrenger, 2006). ATRN mRNA is widely distributed throughout the CNS and is expressed at high levels in regions of the olfactory system, some limbic structures, and regions of the brainstem, cerebellum, and spinal cord (Izawa et al., 2008). ATRN is generally expressed in neurons, but when glioma occurs, the expression of ATRN may also be changed in glial cells (Malík et al., 2001).

ATRN molecular structure is closely related to supporting cell or axon guidance and may play a key role in central nervous system formation and function. Kuramoto et al. (2001) found that in mice mutated with Zitter gene, the vacuoles and myelin of the central nervous system were produced too little, leading to tremors and then local paralysis, which may be accompanied by central nervous system tumors. Thus, the ATRN gene is associated with central nervous system homeostasis. Spatial learning and memory require the formation of myelin, so the spatial memory ability decreases significantly with the reduction of myelin formation during aging. Wang et al. (2020) found that promoting myelin formation could improve the decline of spatial memory function. To date, understanding of the physiological function of ATRN is mainly due to studies on ATRN‐mutant rodents. Zitter rats, originating in a Sprague–Dawley population, were found to be tremor mutants; subsequent genetic analysis showed that the abnormality was caused by mutations in the autosomal recessive gene ATRN (Azouz et al., 2007). The tremors developed spontaneously at 3 weeks of age, and flaccid paresis of the hind limbs was seen at approximately 6 months of age. The main pathological findings were progressive hypomyelination and vacuolization of the central nervous system (CNS) (Bronson et al., 2001). The hypomyelination was characterized by a marked reduction in the density of myelinated fibers and the number of myelin lamellae, accompanied by abnormal or elongated myelination. With increasing age, the vacuoles extended deeper into the cortex, hippocampus, gray matter of the cervical spinal cord, and the granular layer and white matter of the cerebellum, which in turn affected the stability of the CNS (Ehara et al., 2018; Shahrour et al., 2017). Although ATRN has been linked to schizophrenia (Li et al., 2021), its specific role and molecular mechanisms in mental disorders are poorly understood. However, mutations in ATRN may be associated with a decline in spatial memory (Muenchhoff et al., 2016). A previous study in our laboratory also showed that ATRN‐mutant rats experienced diminished long‐term memory in the water maze (Li et al., 2021), but it is unclear whether ATRN mutations affect working memory.

Working memory, a form of short‐term memory, supports the brain's ability to remember things while performing complex tasks. It is not just a simple repetition of what was observed but the interaction and reprocessing of a series of stimuli, including temporary storage and the deletion, encoding, and integration of information. Previous studies have summarized working memory as an interactive mode of behavior and brain function that temporarily holds and manipulates information during a series of cognitive tasks, such as language comprehension, learning, and reasoning (Kent, 2016; Miller et al., 2018). Thus, working memory can be considered the basis and prerequisite for the successful execution of complex behaviors. Further investigation of the effects of ATRN mutations on working memory could provide a better understanding of the role of ATRN in spatial memory.

To examine the relationship between ATRN and working memory, a novel ATRN‐mutant rat generated via the CRISPR/Cas9 system was used to investigate the effect of ATRN gene mutations on working memory in rats. This study also observed changes in myelin basic protein (MBP) expression in the rats. Therefore, our findings suggest corresponding behavioral changes associated with ATRN mutations in rats. The novel ATRN‐G505 C mutant rat can serve as an ideal model for further study of the multiple functions of ATRN.

## 2 MATERIALS AND METHODS

### 2.1 Animals

The rats used in this study were all male and were mated with a generation of heterozygous ATRN rats provided by Nanjing University. Genotypes were identified 2 weeks after birth, and behavioral tests were performed at 60 days. Briefly, transcript 201 of rat ATRN (attractin, Gene ID: 83526) was selected to create the ATRN mutants, and a G505C mutation was introduced into exon 9. Specifically, the 505th amino acid (aa) glycine (G) was altered to cysteine (C); that is, GGC was altered to TGC (Li et al., 2021). A synthetic primer was inserted into introns 8–9 for subsequent genotyping. The animal mutation model was prepared by Nanjing Biomedical Research Institute of Nanjing University (Nanjing, China). Then, ATRN‐G505C(KI/WT) female rats were crossed with (KI/WT) male rats to produce rats with three genotypes: ATRN‐G505C(WT/WT) (*n* = 14), ATRN‐G505C(KI/WT) (*n* = 25), and ATRN‐G505C(KI/KI) (*n* = 5). The above genotypes were identified by polymerase chain reaction (PCR) assays. The primer sequences were 3444‐ATRN‐wt‐tF2 (CAGGACTGTGCACATGAATAG) and 3444‐ATRN‐wt‐tR2 (GAGATACAGAGAGACTAGTGC). All animal procedures were performed in accordance with the National Institutes of Health Guide for the Care and Use of Laboratory Animals (NIH Publications No. 8023, revised 1978) and were approved by the Animal Care and Use Committee of Jiangsu University. All animals were housed under a reversed light–dark cycle (lights on 20:00–8:00) in a room with controlled temperature (21 ± 1°C) and humidity (55 ± 5%) and were allowed free access to food and water, as described in previous studies from our laboratory.

### 2.2 Water maze working memory test

**FIGURE 1:**
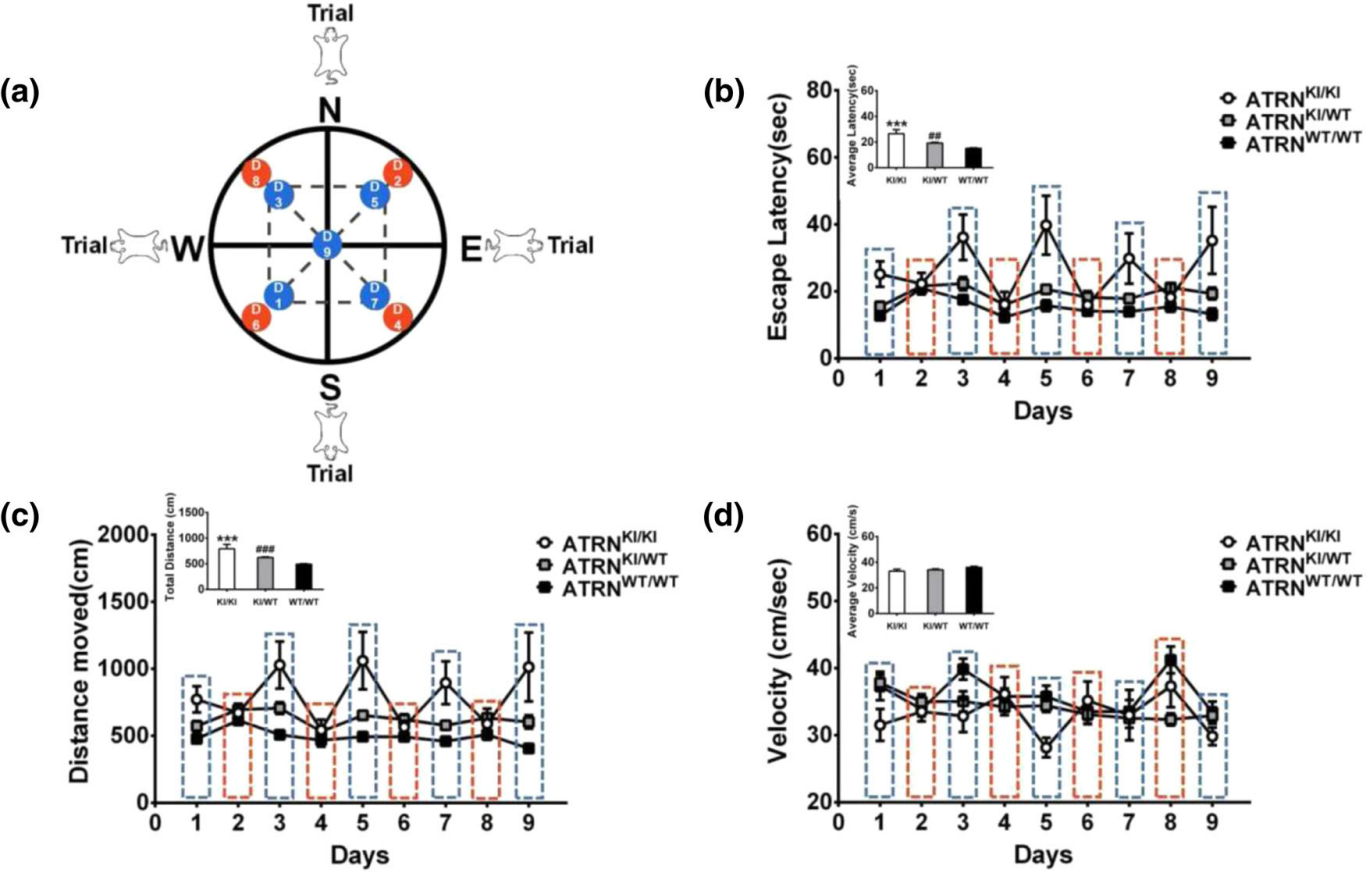
Effect of the ATRN mutation on working memory test. (a) Diagram of the water maze test of working memory. The working memory water maze task consisted of 9 days of acquisition in which the platform location changed every day. (b) The escape latency of rats with different genotypes in the working memory test (*H* = 22.22, *p* < .001). ***p < .001, ATRN^KI/KI^ (*n* = 5) versus ATRN^KI/WT^ (*n* = 25); **##** *p* < .01, ATRN^KI/WT^ (*n* = 25) versus ATRN^WT/WT^ (*n* = 14). (c) The distance traveled by rats with different genotypes in the working memory test (*F* = 24.73, *p* < .001). ***p < .001, ATRN^KI/KI^ (*n* = 5) versus ATRN^KI/WT^ (*n* = 25) **###** *p* < .001, ATRN^KI/WT^ (*n* = 25) versus ATRN^WT/WT^ (*n* = 14). (d) The swimming speed (velocity) of rats with different genotypes in the working memory test (*F* = 2.21, *p* > .05). ATRN, attractin

The maze was a circular pool 160 cm in diameter and 75 cm in height that contained water at 20 ± 5°C. The interior of the swimming pool was painted black, and there was a removable escape platform 10 cm in diameter placed 2 cm below the surface of the water, not visible to a rat in the pool, which provided a base for the rats to stand on (and thereby cease swimming). The working memory water maze task consisted of 9 days of training in which the platform location changed every day (Pouzet et al., 2002), with the same platform position used for all the animals on a given day. For each day of training, the location of the escape platform was fixed, but each of the four trials, a different starting point (N, E, S, or W) was used. Nine platform positions were used, including the middle of the NW, NE, SW, or SE quadrants, either 38 cm from the wall or adjacent to the wall (in the middle between each pair of starting points), and the center of the pool. The starting points for successive trials were different (see Figure 1a for more details).

Rats were given four trials per day. At the beginning of the test, the rats were placed into the water at the starting position. The trial ended when the rat reached the escape platform, and the escape latency was recorded for each trial. If a rat failed to escape within 60 s, the maximum escape latency was recorded as 60 s, and it was guided to the platform by the experimenter. The rat was then allowed to stay on the escape platform for 15 s before being returned to its home cage for 20 s before the start of the next trial, resulting in an intertrial interval of 35 s. After the trials for the day were completed, the rats were dried with a towel and returned to their home cage. Three independent measures, (1) swimming distance (cm), (2) swimming velocity (cm/s), and (3) escape latency (s), were tracked and digitized, and information was stored for subsequent analysis (Pouzet et al., 2002). At the end of the water maze experiment, the brains of the rats were removed within 2 h of the final trial and stored at 4°C for later use.

### 2.3 Western blot analysis

Western blotting and semiquantitative analyses were performed in accordance with previously described procedures (Li et al., 2021). In brief, the rats (*n* = 3 in each group) were decapitated after the behavioral experiments. Hippocampal proteins were immediately frozen in liquid nitrogen and homogenized in radioimmunoprecipitation assay (RIPA) tissue protein extraction reagent (Beyotime, Shanghai, China) containing appropriate protease inhibitors (PMSF, Beyotime, Shanghai, China). The primary antibodies and dilution rates were as follows: MBP (1:1000, Abcam, Cambridge, MA, USA) and β‐tubulin (1:1000, Proteintech Group). Then, horseradish peroxidase‐conjugated secondary antibodies (diluted to 1:5000, Beyotime, Shanghai, China) were applied, and the bands were fixed and visualized by an ECL advanced kit (Beyotime Institute of Biotechnology). β‐Tubulin was utilized as an internal control for protein loading and transfer efficiency. The protein expression was quantified by ImageJ (National Institutes of Health, Bethesda, MD, USA).

### 2.4 Statistical analysis

All statistical analyses were performed with SPSS 22.0 (IBM, Armonk, New York, USA) and GraphPad Prism 6 (Inc, San Diego, CA, USA). One‐way ANOVAs and repeated‐measures ANOVAs were used to determine statistical significance. Post hoc comparisons were conducted with Fisher's protected least significant difference test. The Kruskal–Wallis test was used to evaluate the difference among groups of latency to the platform, and Nemenyi test was used for post hoc test. Data are expressed as the means ± standard error of the mean, except the latency was expressed as the means ± median. The statistical significance level was established at *p* < .05.

## 3 RESULTS

### 3.1 Effect of ATRN mutation on working memory

As shown in Figure 1a, after 9 days of training in the water maze working memory test, there was a significant main effect of genotype: ATRN‐G505C(KI/KI) rats had a significantly longer escape latency (H = 22.22, *p* < .001, Figure 1b) and a large increase in swimming distance (F(2,35) = 24.73, *p* < .001, Figure 1c), but there was no significant difference in swimming speed among the three genotypes (F(2,35) = 2.21, *p* > .05, Figure 1d). These findings suggest that the significant increase in the time and swimming distance required to locate the platform in the ATRN‐G505C(KI/KI) rats was due to spatial memory impairment rather than motor impairment. Moreover, the effect of the Genotype × Escape Latency interaction was significant (ps < .001). Interestingly, escape latency and swimming distance trends over the 9‐day experiment showed that ATRN‐G505C(KI/KI) rats took significantly longer than the other two groups on Days 1, 3, 5, 7, and 9 of the experiment (blue dashed box), while all the measurements of all three groups were similar on Days 2, 4, 6, and 8 (red dashed box). The data points differed when the platform was placed in the middle of a quadrant or at the center of the water maze and were more similar when the platform was placed at the edges. These results suggest that the effect of the ATRN gene mutation on working memory was not homogeneous throughout the test period and might have been influenced by the platform position.

**FIGURE 2:**
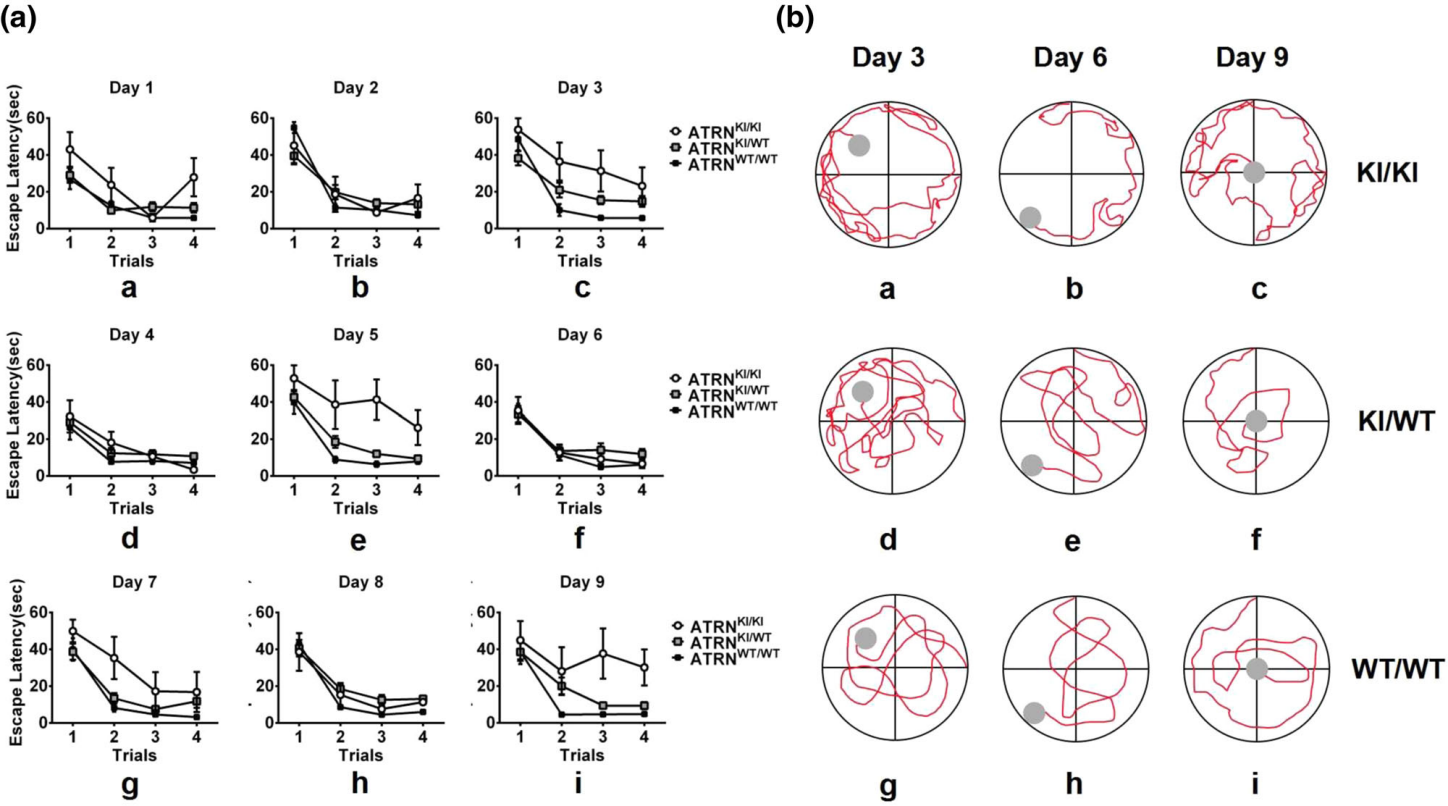
Performance on the working memory test on each day of training (9 days in total) and the representative swimming paths for the three groups on Days 3, 6, and 9. (A) The escape latency of the three groups of rats in the working memory test on Days 1–9. (a,c,e,g,i) The escape latency separately on Day 1 (*H* = 7.78, *p* < .01), Day 3 (*H* = 7.52, *p* < .01), Day 5 (*H* = 9.89, *p* < .01), Day 7 (*H* = 5.55, *p* > .05), and Day 9 (*H* = 6.31, *p* < .05); ATRN^KI/KI^ (*n* = 5) versus ATRN^KI/WT^ (*n* = 25) or ATRN^WT/WT^ (*n* = 14). (b,d,f,h) The escape latency separately on Day 2 (*H* = 0.27, *p* > .05), Day 4 (*H* = 2.47, *p* > .05), Day 6 (*H* = 3.24, *p* > .05), and Day 8 (*H* = 4.57, *p* > .05). (B) The representative swimming paths of the three groups of rats in the working memory test with different platform location on Days 3, 6, and 9. (a–c) The swimming paths of ATRN^KI/KI^ rats. (d–f) The swimming paths of ATRN^KI/WT^ rats. (g–i) The swimming paths of ATRN^WT/WT^ rats. ATRN, attractin

A separate analysis of each day's trials revealed that the escape latency of ATRN‐G505C(KI/KI) rats was still significantly longer than that of ATRN‐G505C(KI/WT) and wild‐type rats when the platform was placed in the middle of a quadrant or in the center of the water maze (Day 1: *H* = 7.78, *p* < .01, Figure 2A(a); Day 3: H = 7.52, *p* < .01, Figure 2A(c); Day 5: H = 9.89, *p* < .01, Figure 2A(e); Day 7: *H* = 5.55, *p* > .05, Figure 2A(g); Day 9: *H* = 6.31, *p* < .05, Figure 2A(i)). However, when the platform was placed at the edge, there was no significant difference in the escape latency among the three genotypes of rats (Day 2: *H* = 0.27, *p* > .05, Figure 2A(b); Day 4: *H* = 2.47, *p* > .05, Figure 2A(d); Day 6: *H* = 3.24, *p* > .05, Figure 2A(f); Day 8: *H* = 4.57, *p* > .05, Figure 2A(h)). Moreover, the trials × genotype interaction was not significant on each day (all *p*_s_> .05). Taken together, these findings suggest that the different escape latencies of the three groups were most likely related to the differences in genotype.

We further compared the swimming trajectories of rats in each group with different platform positions. We found that ATRN‐G505C(KI/WT) and wild‐type rats dynamically altered their swimming paths according to the platform locations to rapidly reach the platform; however, the search strategy of the ATRN‐G505C(KI/KI) rats appeared to be static over the 9‐day experiment (Figure 2B). When these rats were placed in the water, they first sought the platform along the edge of the pool and only then ventured into the middle region; exploration of the edge of the pool often consumed a substantial amount of time. Therefore, these results suggest that ATRN‐G505C(KI/KI) rats took longer to find the platform when it was not located at the edge because of a failure to update learning strategies or working memory and not because of spatial memory impairments. Thus, ATRN gene mutations could cause working memory impairments.

### 3.2 Effect of the ATRN mutation on MBP expression in the brain

**FIGURE 3:**
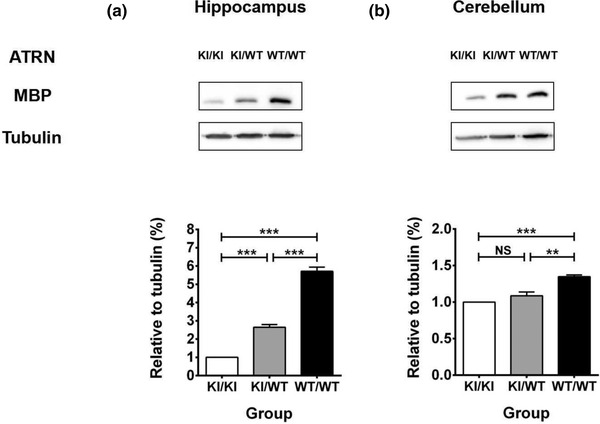
Effect of the ATRN mutation on MBP expression in the hippocampus and cerebellum. (a) The expression of MBP in the hippocampus among the different genotypes. F(2,8) = 232.26, *p* < .001; *** *p* < .001. *n* = 3 for each group. (b) The expression of MBP in the cerebellum among the different genotypes. F(2,8) = 27.87, *p* < .01; ** *p* < .01; NS, no significance. *n* = 3 for each group. ATRN, attractin

The effect of the ATRN mutation on CNS homeostasis was examined by comparing the expression of MBP in the hippocampus and cerebellum among the three groups of rats. We found that MBP levels in the hippocampus (Figure 3a) were significantly decreased in ATRN‐G505C(KI/KI) and ATRN‐G505C(KI/WT) rats compared with wild‐type rats (WT/WT, F(2,8) = 232.26, *p* < .001) and that this decrease was even more significant in ATRN‐G505C(KI/KI) rats than in ATRN‐G505C(KI/WT) rats (*p* < .001). Consistent with this finding, the expression of MBP was significantly reduced in the cerebellum of ATRN‐G505C(KI/KI) and ATRN‐G505C(KI/WT) rats compared to that of wild‐type rats (WT/WT, Figure 3b) (F(2,8) = 27.87, *p* < .01), but the expression did not significantly differ between these two groups (*p* > .05). This confirmed that ATRN mutations suppress MBP expression in the brain and may thereby affect CNS function.

## 4 DISCUSSION

In this study, the ATRN homozygous mutant rats performed worse than the ATRN heterozygous mutant rats: They did not seem to develop correct spatial perception despite repeated training in the water maze working memory test, although the heterozygous rats formed somewhat reliable memories of the platform location during this time. Although heterozygous rats did not perform as well as the wild‐type rats, we concluded that the working memory impairment due to heterozygous mutation in ATRN was not as severe as that caused by homozygous mutations; this could indicate that partial mutations in ATRN do not completely disrupt spatial memory or that other compensatory mechanisms are employed. Additionally, the reduced expression of MBP in ATRN homozygous rats suggests that the stability of myelin structure and function in the CNS is disrupted, which may trigger the disruption of spatial perception and memory seen in this mutation.

The ATRN gene is related to pigmentation, central nervous system, and immune regulation. Therefore, the mutation of ATRN gene will affect the homeostasis of central nervous system. Zitter rats developed tremor at 3 weeks of age and flaconic hind limb paralysis at about 6 months of age. The main pathological manifestations were progressive myelotopia and vacuolization of the central nervous system (Kuramoto et al., 2001). The initiation of myelination and the basic structure of the myelin sheath are normal, but over time, the density of myelin fibers and layers is abnormally low, as well as abnormal or elongated myelination. The splice site at the 12th exon‐intron junction of the ATRN gene was mutated to be homozygous. Clinical and neuropathological phenotypes were saved by expressing the WT membrane form of ATRN instead of the soluble form. Another ATRN spontaneous rodent mutant, the black tremor hamster, is characterized by tremors in the trunk and back legs secondary to the abnormally thin myelin sheath of all central axons (Nunoya et al., 1985). When 10 kb is inserted into ATRN, the molecule is homozygous, resulting in complete loss of the membrane isoform (Kuramoto et al., 2002). Finally, several mouse ATRN mutants were characterized by fur color changes and neurodegenerative changes (Bronson et al., 2001; Gunn et al., 2001). ATRN mg‐6J mice with the deletion of most of the ATRN gene (exon 1–27) genome had mahogany‐colored coat, extended gait, and tremors. Dyskinesia can be severe, affecting the extent of feeding, and some mice have died by 3–4 weeks of age. Seizures observed in some ATRN mg‐3J homozygous mice are characterized by sudden freezing of motion followed by rollover. Beginning at 2 months, the mutant developed severe vacuolation of the brain, brainstem, cerebellar granular layer, and spinal cord, which severely affected myelination in the central nervous system.

In this study, experimental manipulation and individual animal differences, as well as the test environment, will affect the experimental results. Therefore, during the experiment, blind detection was used to minimize the effects of experimenter factors, that is, the analysis participants were different from the actual participants, who did not know the specific group of rats. Of course, in order to avoid large individual differences between rats, it is necessary to ensure the unity of the breeding environment, including the fact that the breeder and the actual experimental operator must be the same person, to prevent the distortion of the experimental results caused by the stress of the rats on the experimental personnel during the experiment. In terms of experimental environment, in addition to ensuring appropriate water temperature and light intensity, researchers should also try to avoid interference to rats. Therefore, during the experimental process, the researchers should control the time whether they put the rats in the water or guide the rats on the platform.

Due to spatial and temporal resolution limitations in previous studies, only estimates of the highest density of the most active neural elements (cells and fibers) in brain regions involved in spatial memory representations, whether sensory, motor, emotional, or associative, could be calculated. However, these methods are clearly insufficient for characterization of the fine‐grained details and distribution of neural elements involved in specific memories. Recent studies have revealed the complexity of the structural connectivity between brain areas underlying these memories (Brincat et al., 2018; Reinert et al., 2021). However, the directional and regulatory mechanisms responsible for the functional interactions underlying the specific content of these memories remain unclear. The hierarchical, analog, and probabilistic nature of interactions in the neurocognitive domain makes the study of this issue even more complex. A new paradigm is therefore needed to help understand the microstructure and dynamics of spatial memory and cognition.

Reduced levels of MBP in ATRN homozygous mutants are a hallmark of myelin degeneration and impaired CNS homeostasis. Myelin disruption and impaired myelin repair, indicated by reduced densities of oligodendrocyte precursors, oligodendrocytes, and myelin, are frequently observed in the brains of Alzheimer's disease patients and of various mouse models (Zhan et al., 2018). Liu's study confirmed that treatment with 2% w/w phosphatidylserine emulsion for 3 months enhanced spatial learning and memory in the Morris water maze test in 5‐ and 12‐week‐old mice. Western blot analysis showed that this was mainly due to the upregulation of BDNF, TrkB, PSD95, mTOR, MBP, and ErbB4 expression by phosphatidylserine in the mouse hippocampus. In addition, reverse transcription‐polymerase chain reaction (RT–PCR) analysis revealed elevated Nrg‐1 and ErbB4 mRNA expression in the phosphatidylserine emulsion‐treated group; high Nrg‐1 and ErbB4 expression levels are associated with better myelination (Liu et al., 2022), while Nrg‐1 gene knockdown in mice lead to schizophrenia‐like behavior (Cong et al., 2022). These results imply that ATRN mutations may reduce myelination and impair learning and memory in rats by inhibiting BDNF/TrkB and Nrg‐1/ErbB4 signaling. ATRN is important for myelination, so ATRN may be added to the growing list of genes associated with low myelin leukodystrophy, which seems to be limited to the central nervous system, so ATRN may be a new clinical breakthrough in the study of this disease. The findings of the present study are also consistent with previous studies in our lab (Li et al., 2021), suggesting the novel ATRN mutant rat can serve as an ideal model for further study of the multiple functions of ATRN.

## CONFLICT OF INTEREST

The authors have no conflict of interest to declare.

### PEER REVIEW

The peer review history for this article is available at https://publons.com/publon/10.1002/brb3.2876.

## Data Availability

The data that support the findings of this study are available from the corresponding author upon reasonable request.
